# The presence of De Winter electrocardiogram pattern following elective percutaneous coronary intervention in a patient without coronary artery occlusion: A case report

**DOI:** 10.1097/MD.0000000000018656

**Published:** 2020-01-31

**Authors:** Shi Chen, Hua Wang, Liwei Huang

**Affiliations:** a Department of Cardiology, West China Hospital, Sichuan University; b Ultrasound in Cardiac Electrophysiology and Biomechanics Key Laboratory of Sichuan Province, Cardiovascular Ultrasound and Non-invasive Cardiology Department, Affiliated Hospital of University of Electronic Science and Technology of China, Sichuan Academy of Medical Sciences, Sichuan Provincial People's Hospital, Chengdu, Sichuan, P.R. China.

**Keywords:** De Winter electrocardiogram pattern, electrocardiogram, percutaneous coronary intervention, perioperative myocardial infarction

## Abstract

**Rationale:**

The De Winter electrocardiogram (ECG) pattern is considered as a ST elevated myocardial infarction (STEMI)-equivalent pattern. Due to its rare nature, it is unclear whether this ECG pattern suggests the presence of some other condition.

**Patient concerns:**

We reported a 47-year-old man with new-onset chest discomfort several hours after the second-stage percutaneous coronary intervention (PCI).

**Diagnoses:**

An emergency coronary angiogram (CAG) did not show any abnormality. However, the dynamic changes in the ECG and myocardial biomarkers indicated perioperative myocardial infarction.

**Intervention:**

The patient was monitored in the cardiac care unite (CCU), and was administered an intravenous infusion of diltiazem and subcutaneous injection of low molecular weight heparin.

**Outcomes:**

After a few hours, his symptoms were alleviated. The patient was discharged after 6 days of hospitalization without any complications.

**Lessons:**

The De Winter ECG pattern can be observed in patients without significantly coronary arteries occlusion. The newly onset De Winter ECG pattern after PCI procedure may indicate perioperative myocardial infarction caused by impaired microvascular perfusion.

## 1 Introduction

Recent studies have found that certain patients with an acute thrombotic occlusion of a large epicardial coronary artery do not show ST-elevation on the standard 12-lead electrocardiogram (ECG).^[1]^ This unusual De Winter ECG pattern manifests as ST-segment depression in leads V1 to V6, accompanied by tall, positive symmetrical T waves.^[2]^ Thus far, nearly all the reported cases with this ECG pattern have exhibited proximal left anterior descending (LAD) artery occlusion.^[2,3]^ Therefore, the De Winter ECG pattern is considered as a ST elevated myocardial infarction (STEMI)-equivalent pattern. Due to its rare nature, it is unclear whether this ECG pattern suggests the presence of some other condition. In the present report, we describe the case of a STEMI patient who underwent second-stage percutaneous coronary intervention (PCI) and subsequently developed a perioperative myocardial infarction that presented a De Winter ECG pattern, despite the coronary angiogram (CAG) not showing occlusion in any arteries.

## 2 Case report

**Figure 1:**
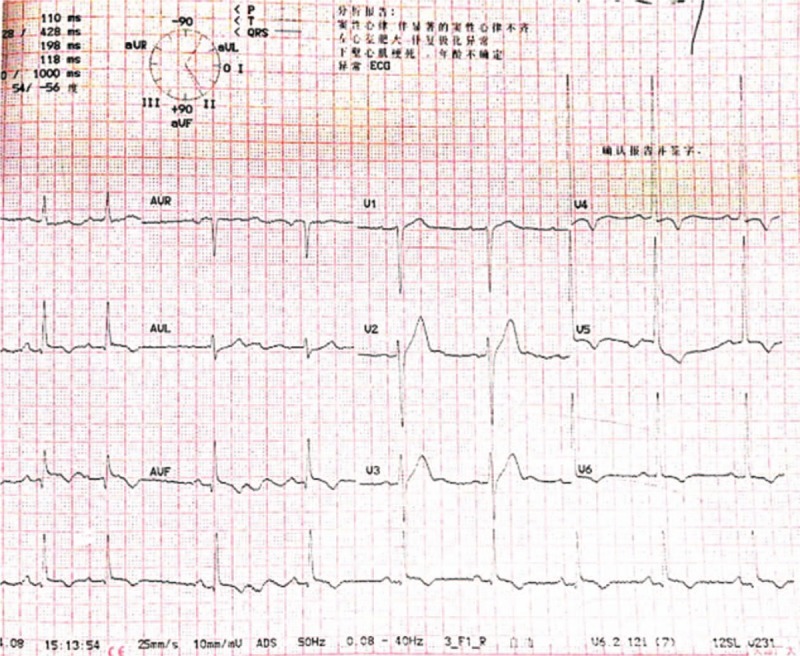
Initial ECG showing mild ST-segment elevation in the inferior leads (II, III, and aVF). ECG = electrocardiogram.

**Figure 2:**
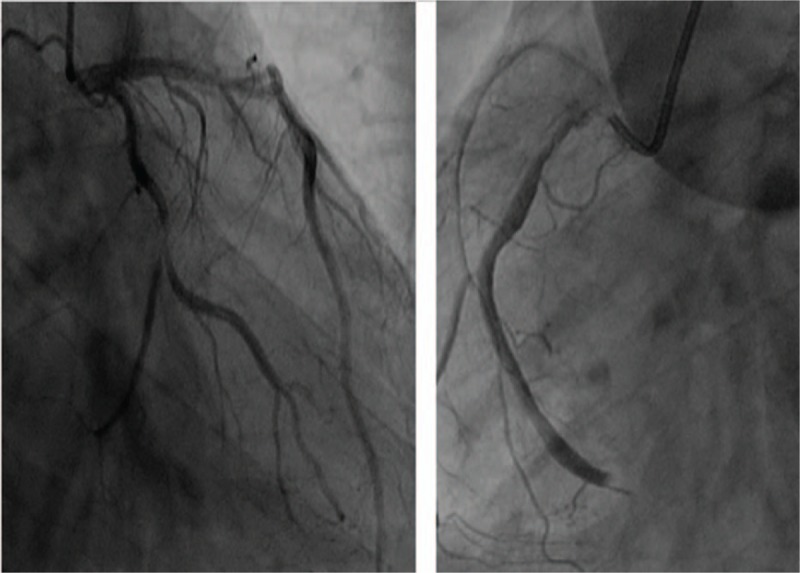
Emergency coronary angiography showing total occlusion in the distal segment of the right coronary artery (RCA), and severe stenosis in the middle segments of the left anterior descending (LAD) artery and left circumflex artery (LCX).

**Figure 3:**
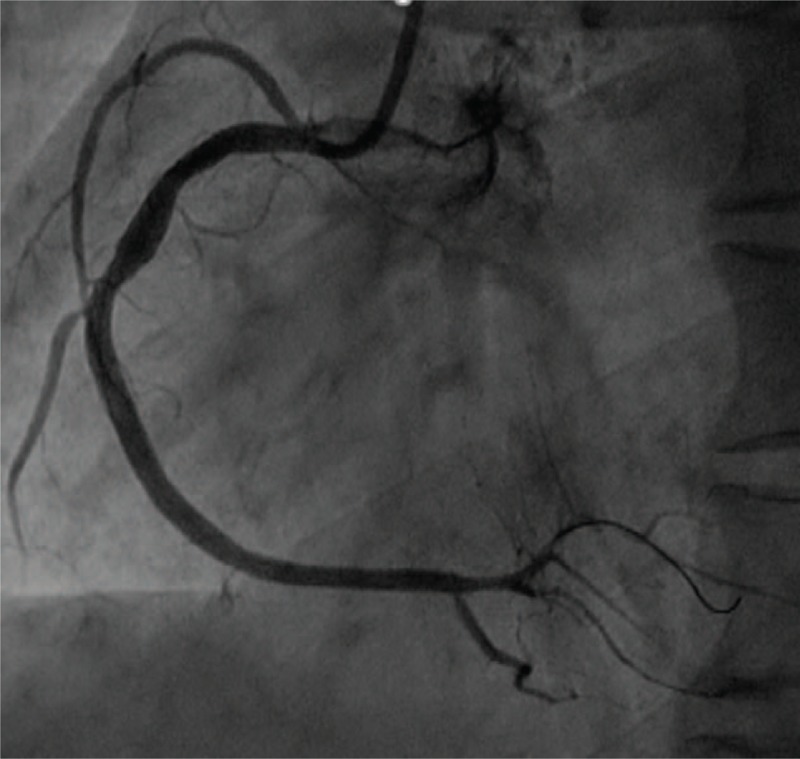
Coronary angiography after emergency PCI showing a very well developed right coronary artery with TIMI-3 blood flow. PCI = percutaneous coronary intervention.

**Figure 4:**
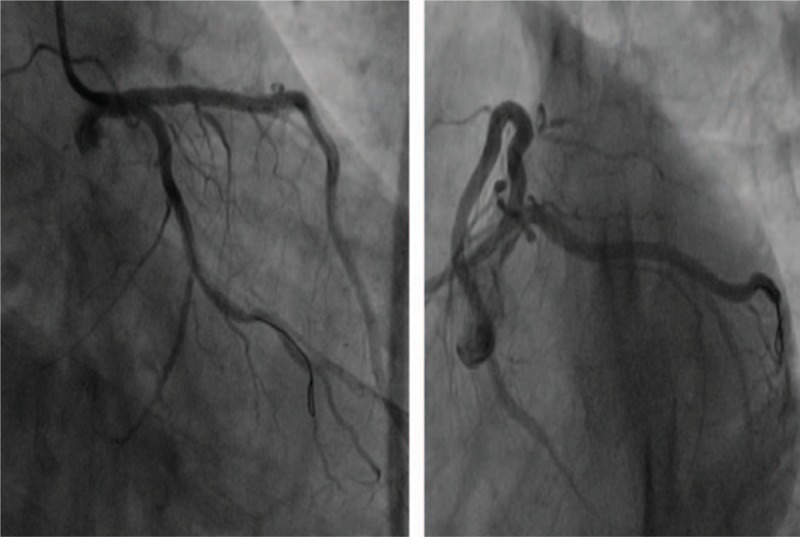
Coronary angiography after second-stage PCI showing a very good blood flow in the left anterior descending (LAD) artery and left circumflex artery (LCX). PCI = percutaneous coronary intervention.

**Figure 5:**
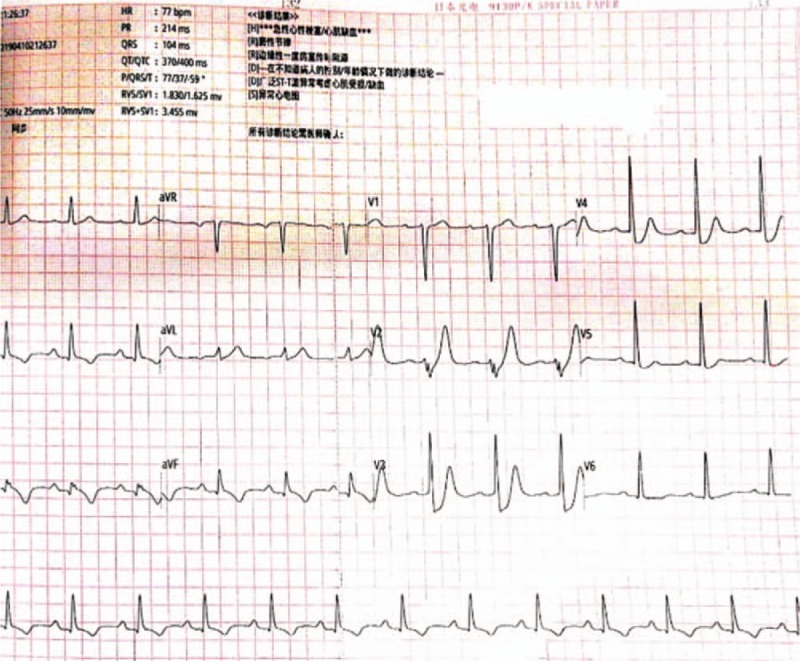
Post-procedural ECG showing junctional ST-depression and tall symmetrical T-waves in leads V2–4. ECG = electrocardiogram.

A 47-year-old man with a history of hypertension was admitted to the emergency department for typical acute chest pain nearly 18 hours after symptom onset. In the emergency department, an ECG (Fig. 1) was immediately obtained, which showed a sinus rhythm of 60 bpm; inverted T waves in leads V4–6, II, III, and aVF; abnormal Q waves; and mild ST segment elevation in leads II, III, and aVF, suggesting an inferior myocardial infarction. His troponin T and creatine kinase-myocardial band (CK-MB) levels were 1968 ng/L (normal range, 0–14 ng/L) and 199.6 ng/mL (normal range, <4.94 ng/mL), respectively. Initial management included the administration of aspirin (300 mg), clopidogrel (300 mg), and atorvastatin (20 mg). The patient was then immediately transferred to our cardiac catheterization lab. Emergency CAG indicated multi-vessel disease, including a culprit lesion in the distal segment of the right coronary artery (RCA) that was totally occluded, as well as severe stenosis in the middle segments of the LAD artery and left circumflex artery (LCX; Fig. 2). However, we decided to treat only the culprit vessel. Accordingly, primary PCI was successfully performed, and a 2.75 × 24 mm drug-eluting stent was placed in the RCA with the final flow recorded as TIMI-3 (Fig. 3). After 2 days, a second-stage procedure was performed, and 2 drug-eluting stents (3.0 × 24 mm and 2.75 × 29 mm, respectively) were placed in the LAD and LCX with good angiographic results (Fig. 4). At the end of the operation, the patient experienced some chest discomfort, and was transferred to the cardiac care unit (CCU). Post-procedural ECG showed T-wave inversion in leads II, III, and aVF. After 2 hours, the patient complained of a new-onset chest discomfort and left shoulder pain. ECG (Fig. 5) showed a sinus rhythm of 77 bpm, as well as junctional ST-depression with tall symmetrical T-waves in leads V2–4.

**Figure 6:**
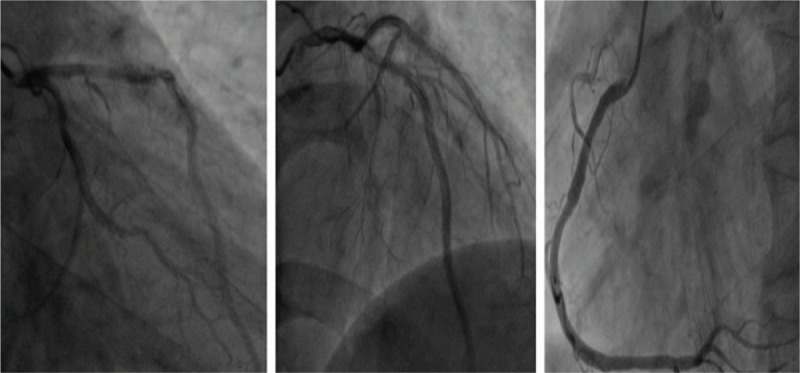
Emergency coronary angiography showing TIMI-3 flow in the left anterior descending (LAD) artery, left circumflex artery (LCX), and right coronary artery (RCA) without any in-stent thrombosis or main side branch occlusion.

**Figure 7:**
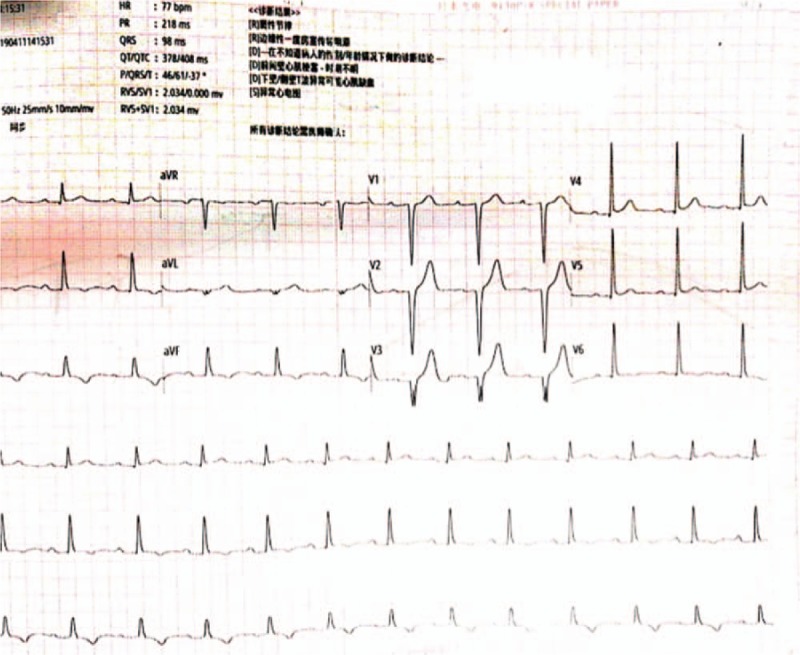
ECG showing a deepened Q wave with resolution of ST-segment elevation in the precordial leads. ECG = electrocardiogram.

With regard to myocardial enzyme levels, his CK-MB and troponin T values were 23.56 ng/mL and 3007 ng/L, respectively. We believed that in-stent thrombosis and main side branch occlusion could not be excluded. Hence, emergency CAG was performed, but no significant abnormality was noted (Fig. 6). The patient was then monitored in the CCU, and was administered an intravenous infusion of diltiazem (15 mg/h) for 24 hours and subcutaneous injection of low molecular weight heparin (4000 IU/d) for 3 days. After a few hours, his symptoms were alleviated. On the next morning, the patient did not report any chest discomfort or shoulder pain. However, his cardiac biomarker levels remained significantly elevated (CK-MB: 134.7 ng/mL; troponin T: 3304 ng/L). ECG (Fig. 7) indicated a deepened Q wave with resolution of the ST-segment elevation in the precordial leads. The patient was discharged after 6 days of hospitalization without any complications but the cardiac biomarkers were still elevated (CK-MB: 42.3 ng/mL; troponin T: 1209 ng/L) and echocardiography showed dyskinesis in the anterolateral segment with a normal left ventricular ejection fraction. The patient is currently being followed up by the cardiology department.

## 3 Discussion

In 2008, De Winter first described a special ECG pattern wherein the upward-sloping ST-segment depression in leads V1 to V6 was accompanied by tall, positive symmetrical T waves.^[2]^ He detected this ECG pattern in 2% of patients with LAD occlusion, and believed that it was a STEMI-equivalent pattern. In another larger group of 5588 ACS patients, de Winter et al^[3]^ identified 11 patients with the De Winter ECG pattern. These 11 angiograms invariably indicated the involvement of the proximal LAD. All the patients received PCI but 3 patients (27%) died within the first week.

The electrophysiological explanation of why these patients did not show ST-segment elevation in the presence of an occluded proximal LAD artery remains elusive. Verouden et al^[4]^ suggested that the area of transmural ischemia was large, and hence, no injury currents were generated towards the precordial leads but were only directed upwards to the standard lead aVR. Theoretically, an anatomical variant of the Purkinje fibers with endocardial conduction delay could also result in this special ECG pattern. Some researchers believe that the absence of ST-segment elevation may be related to the lack of activation of sarcolemmal adenosine triphosphate (ATP)-sensitive potassium (KATP) channels by ischemic ATP depletion, as has been found in KATP-knockout animal models of acute ischemia.^[5]^

As cases with the De Winter ECG pattern are rare, it is currently unclear whether it is also observed in other clinical conditions besides myocardial infarction due to LAD occlusion. This specific ECG pattern has recently been described in acute myocardial infarction patients with a culprit lesion in the first diagonal branch^[6]^ or RCA.^[7]^ Moreover, Xu et al^[8]^ described a case with the De Winter ECG pattern wherein emergency CAG confirmed the occlusion of the obtuse marginal artery. A systemic review indicated that this ECG pattern may be observed in cases with either LAD or LCX occlusion.^[9]^

To our knowledge, this is the first case exhibiting the De Winter ECG pattern following PCI. Emergency CAG showed TIMI-3 blood flow in both the left and right coronary arteries, and in-stent thrombosis or main side branch occlusion were excluded. Nevertheless, the dynamic changes in the ECG and cardiac biomarkers indicated the presence of perioperative myocardial infarction. We believe that acute impairment of microvascular reperfusion might have caused this condition.^[10]^

## Author contributions

**Conceptualization:** Hua Wang, Shi Chen.

**Investigation:** Shi Chen.

**Project administration:** Shi Chen.

**Supervision:** Hua Wang.

**Validation:** Hua Wang.

**Writing – original draft:** Shi Chen, Liwei Huang.
